# Impact of Residual Zwitterionic Surfactants on Topside Water–Oil Separation of Pre-Salt Light Crude Oil Emulsions

**DOI:** 10.1021/acsomega.5c07388

**Published:** 2025-10-14

**Authors:** Bruno G. Alvarenga, Angela C. P. Duncke, Aurora Pérez-Gramatges, Ana M. Percebom

**Affiliations:** † Laboratory of Surfactant Physical-Chemistry (LASURF), Pontifical Catholic University of Rio de Janeiro, PUC-Rio, Rio de Janeiro, Rio de Janeiro 22451-900, Brazil; ‡ Department of Chemistry, 28099 Pontifical Catholic University of Rio de Janeiro, PUC-Rio, Rio de Janeiro, Rio de Janeiro 22451-900, Brazil

## Abstract

Zwitterionic surfactants have been used as foaming agents in Enhanced Oil Recovery due to their foamability, tolerance to reservoir conditions, low toxicity, and low adsorption on reservoir rocks. However, in the case of back-production, the impact of zwitterionic surfactants on water-in-crude oil (W/O) emulsions and produced water remains unclear, representing a knowledge gap in the field. The present study evaluated the effect of three commercial zwitterionic surfactants, at concentrations ranging from 50 to 1000 ppm, on water–oil separation using a Brazilian Pre-Salt crude oil. Emulsion formation, separation kinetics, and water quality were assessed through bottle tests, droplet size microscopy, and oil-in-water content analysis. At the lowest concentration (50 ppm), these surfactants promoted coalescence and sedimentation of W/O emulsions faster than traditional nonionic and anionic surfactants. Separation kinetics increased as surfactant concentration increased up to 200 ppm, but concentrations above 500 ppm induced the formation of multiple emulsions (W/O/W). After 72 h, these emulsions separated, and the aqueous phase still contained residual oil droplets, indicating potential challenges associated with the disposal or reuse of produced water. Surfactant hydrophobicity, reduction in interfacial tension, and critical micelle concentration were critical properties influencing the observed effects. The surfactant with higher hydrophobicity induced a more significant and rapid reduction in interfacial tension, resulting in faster W/O separation at low concentrations but compromising water quality at high concentrations. These results indicate that careful selection and monitoring of surfactants during EOR operations can optimize oil recovery efficiency while minimizing operational and environmental risks upstream.

## Graphical abstract

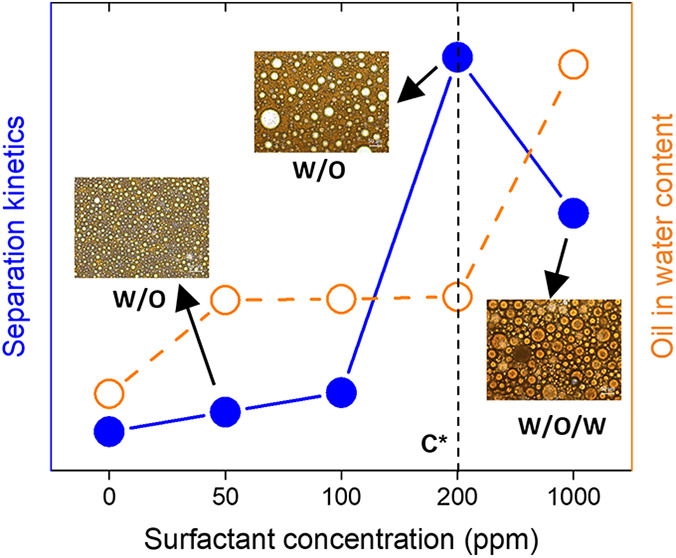

## 1 Introduction

Is the foaming ability of zwitterionic surfactants a double-edged sword in the separation of water/crude oil emulsions? Zwitterionic surfactants can be used as foaming agents to Foam Assisted Water Alternating Gas (FAWAG) for Enhanced Oil Recovery (EOR)^1–8^ due to their desirable interfacial properties, chemical stability, good foamability, salt and temperature tolerance, biodegradability, and low toxicity.^3^ Moreover, these molecules exhibit low adsorption on reservoir rocks, including on the positively charged carbonate surfaces, attributed to their electrically neutral character resulting from the simultaneous presence of positive and negative charges in the polar headgroup.^9,10^ Although reduced surfactant adsorption facilitates their propagation through the reservoir, surfactant back-production could negatively affect upstream processes, notably the separation of water-in-crude oil (W/O) emulsions stabilized by indigenous interfacially active oil compounds.^11,12^ This issue has both industrial and environmental implications. Inefficient oil–water separation increases operational costs in production facilities^13^ and compromises upstream processing,^14^ while dispersed oil in produced water represents a major environmental concern due to its toxicity and persistence, requiring effective treatment to meet increasingly strict discharge regulations.^15^ Thus, understanding how zwitterionic surfactants affect emulsion formation and stability in crude oil–brine systems is crucial to prevent operational complications during oil production.

The concentration of foaming surfactants injected in FAWAG operations typically ranges from 1000 to 50,000 ppm, depending on reservoir conditions, surfactant type, rock properties, and operational objectives.^16–19^ Laboratory experiments have shown that surfactant breakthrough in produced water can vary between 5% and 30% of the injected concentration in porous media,^16,20^ resulting in final concentrations in produced fluids from 50 to 15,000 ppm. Within this concentration range, studies involving anionic,^12,21,22^ cationic,^23–25^ nonionic surfactants,^21,26–30^ biosurfactants,^31^ and surfactant mixtures with other chemicals as alcohol^32^ or polymers,^33^ indicate that these surfactants can either promote water–oil separation or complicate it, potentially impairing the quality of separated water.^12,23,24^ Key factors affecting this outcome include solubility of surfactant in aqueous and oily phase,^22^ pH,^21^ colloidal and interfacial properties of the surfactants, Hydrophilic–Lipophilic Balance (HLB),^22,27^ along with the salinity^21^ of the aqueous phase and the properties of the crude oil.^24,26^

Despite the potential use of zwitterionic surfactants in FAWAG-EOR,^3^ the specific effects of betaine-based surfactants on crude oil emulsions remain largely unexplored, even though they are among the most promising candidates for such applications. Betaine surfactants have highly polar, neutral head groups that promote strong molecular interactions, resulting in both low critical micelle concentration (CMC) and high interfacial activity compared to ionic surfactants with similar hydrocarbon tail lengths.^34–36^ Furthermore, betaine surfactants form elastic and stable interfacial films^37,38^ and are highly water-soluble, properties that, according to the Bancroft Rule, could drive W/O emulsions to invert into oil-in-water (O/W) emulsions. Consequently, although betaine surfactants tend to destabilize W/O emulsions, they could paradoxically stabilize O/W emulsions in the separated water phase, negatively impacting water quality. These features enable betaine surfactants to affect emulsion stability even at residual concentrations, highlighting the importance of investigating their role in water–oil separation and the potential operational implications for produced-water management.

In this context, the present study offers a systematic evaluation of betaine surfactants and their effects on the formation and stability of light crude oil emulsions at residual concentrations. Two betaine surfactants (with varying alkyl chain lengths) and one sulfobetaine (with a different polar headgroup) were compared to traditional nonionic and anionic surfactants. The effects of increasing surfactant concentration (50 to 1000 ppm, considering possible low reservoir adsorption scenarios or overdosing) on emulsion formation, oil–water separation kinetics, and water quality were investigated. Emulsions were prepared with synthetic seawater brine, representative of offshore conditions, and a light Brazilian Pre-Salt crude oil in the presence of surfactants. Emulsion formation and stability, as well as water quality, were evaluated through bottle tests, microscopy (droplet size), and oil-in-water content measurements. The novelty of this work lies in correlating betaine surfactant properties, such as CMC and interfacial tension, with emulsion stability outcomes, highlighting operational and environmental implications for FAWAG-EOR operations.

## 2 Materials and Methods

### 2.1 Materials

A typical Brazilian Pre-Salt light crude oil (Shell, Brazil) was used as received (properties detailed in Table S1). Brine was prepared with NaCl (28.38 g·L^–1^), KCl (0.75 g·L^–1^), CaCl_2_.2H_2_O (0.52 g·L^–1^), MgCl_2_.6H_2_O (1.42 g·L^–1^), and Na_2_SO_4_ (0.035 g·L^–1^). All brine salts, chloroform, and sodium sulfate (for oil extraction) were purchased from Sigma-Aldrich and used without further purification. Five commercial surfactants are listed in Table 1 and were used as received.

**Table 1:**
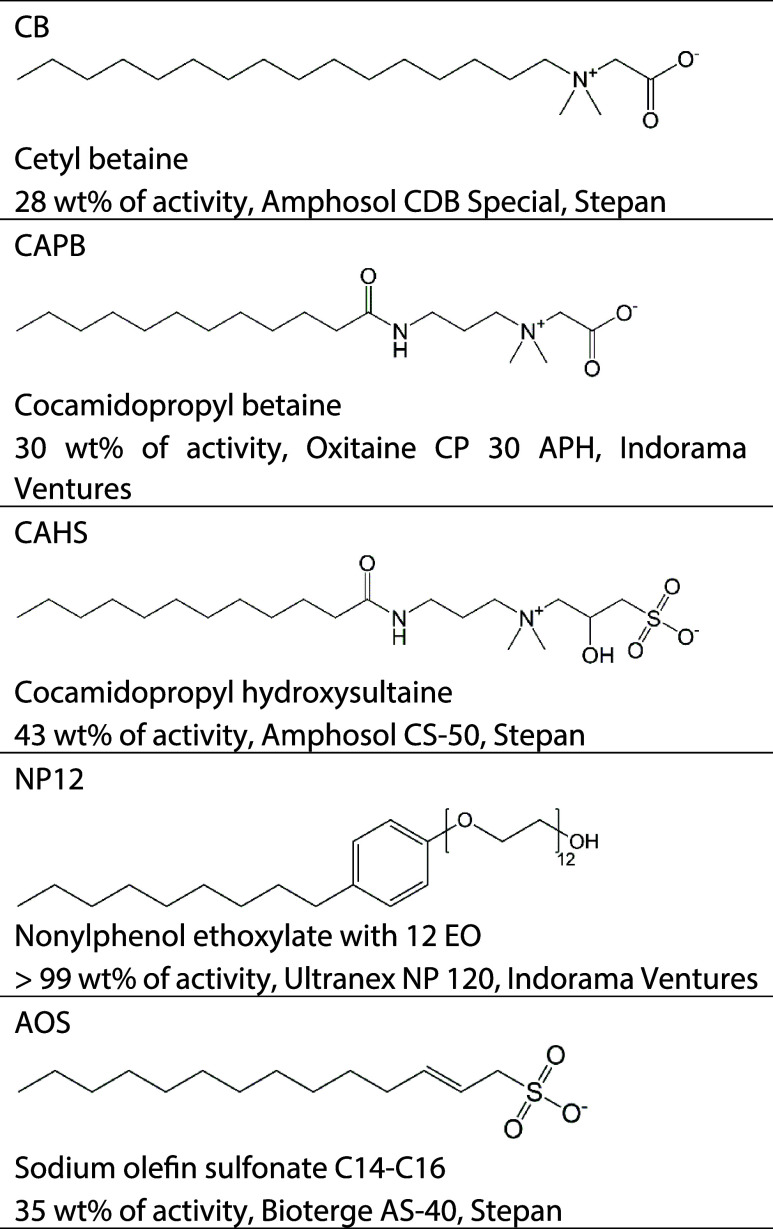
Molecular Structure, Abbreviation, Main Compound Activity, Commercial Name, and Supplier of Commercial Surfactants

### 2.2 Characterization of Surfactants

#### 2.2.1 Critical Micelle Concentration

The air–liquid surface tension (γ) values of different concentrations of the main active compound of the commercial formulations in brine were measured in duplicate using an EZ Pi+ tensiometer (Kibron, Finland) by the platinum rod method at 25 °C. The CMC value for each surfactant in brine was determined by the discontinuity in the γ graph against the logarithm of surfactant concentration, as Rosen and Kunjappu^39^ described.

#### 2.2.2 Interfacial Tension

Dynamic interfacial tension (IFT) was measured using the pendant drop method with a Drop Shape Analyzer 25 tensiometer (Krüss, Germany), applying the Young–Laplace equation.^40^ The oil–brine IFT was determined using a 40 μL oil drop in an aqueous solution containing either 0 or 5 ppm surfactant in brine at 25 °C. Measurements were conducted in duplicate using independent solutions. Due to the rapid IFT reduction and oil droplet detachment, the IFT value after 5 min was selected for calculating the difference (ΔIFT) between crude oil and brine solutions with and without surfactants. This ΔIFT was used to compare the surfactants, regardless of whether equilibrium was reached.

### 2.3 Emulsions

#### 2.3.1 Preparation

Emulsions were prepared by placing 12.5 mL (50 vol %) of crude oil and 12.5 mL (50 vol %) of aqueous phase containing different concentrations of surfactant into 25 mL graduated glass bottles with screw caps. The bottles were heated at 65 °C for 60 min to simulate the reservoir temperature. Afterward, they were manually shaken 10 times with 180° movements and homogenized at room temperature for 2 min at 16,800 rpm, turning the bottle counterclockwise once per second, using a T10 Ultra Turrax with a 10G disperser (IKA, Germany). The bottles were then placed in a water thermal bath at 40 °C for 15 min to reach a temperature similar to that in the gravitational separator.

#### 2.3.2 Stability

The bottles containing the emulsions were maintained at 40 °C for 72 h to evaluate the emulsion stability over time. During this period, images were captured at 0, 5, 10, 15, 30, 60, 120, 1440 (24 h), 2880 (48 h), and 4320 min (72 h) to determine the volume fraction of the released aqueous phase.

#### 2.3.3 Characterization

The emulsion type and droplet size were evaluated using brightfield microscopy with a 20x objective lens on a DM-2700-P microscope equipped with a digital camera MC-190-HD (Leica, Germany). The average droplet size (*D*_1,0_) was determined by measuring 100 droplets using the image processing software ImageJ (Fiji). The polydispersity index (PDI) was calculated using eq 1, where σ^2^ is the variance of the droplet diameters.

1 $$\mathrm{PDI}=\frac{\sigma^{2}}{D_{1,0}^{2}}$$

The water content in oily phases was measured via Karl Fisher titration using the automatic titrator Excellence T9 (Mettler Toledo, Switzerland), with titrations performed in triplicate.

Viscosity measurements were performed at 40 °C and a shear rate of 1 s^–1^ using a Haake Mars 60 rheometer (Thermo Scientific) equipped with a double-gap geometry. A cap was employed to minimize evaporation. The viscosity of fresh emulsions, formed by crude oil–brine and crude oil with 50 ppm of CAHS, was monitored during 2 h.

The conductivity values of selected fresh emulsions were measured using a platinum electrode connected to a 914 conductometer (Metrohm, Switzerland).

### 2.4 Water Quality

After 72 h of the stability test, the aqueous phase was collected, its volume was measured, and it was transferred to a separation funnel. The oil content in the aqueous phase was extracted using chloroform. The obtained chloroform solution was dried using sodium sulfate, and its absorbance was measured at 400 nm using a Cary 60 Spectrophotometer (Agilent Technologies). The oil content was determined using a calibration curve in the concentration range of 30–100 ppm of oil in chloroform (Figure S1). In the case of absorbance saturation, a proper dilution of the samples was carried out to fit the calibration curve. In the absence of surfactants, this analysis was carried out using a different volume proportion of brine in oil (80:20).

## 3 Results and Discussion

### 3.1 Impact of Different Surfactants at Residual Concentrations on the Emulsion Formation and Stability

Because surfactants can be back-produced at residual levels during FAWAG-EOR, their influence on emulsion formation and separation was assessed. The emulsions formed at 50 ppm surfactants were identified as W/O emulsions (Figure 1a). The same type of emulsion was observed even without added surfactants, indicating that indigenous interfacially active oil compounds are the main responsible for emulsification.^41,42^ Initially, at *t* = 0 min, the appearance of the samples and droplet size distributions were similar in all cases (curves of droplet size distribution are shown in Figure S2). Therefore, at 50 ppm (a concentration close to that estimated for back-produced water), the added surfactants had no impact on the emulsion formation. However, while the W/O emulsion without surfactants showed no free water even after 72 h, the emulsions containing 50 ppm of surfactants started to exhibit phase separation after 24 h, which progressively increased over time (48 and 72 h) (Figure 1b). Betaine-based surfactants had a greater effect in destabilizing the emulsions than the nonionic and anionic surfactants, following the order CB ≫ CAPB > CAHS. The destabilization effect was particularly pronounced for CB, which induced an aqueous phase separation within the first minutes and near-complete separation after 72 h. Although these results do not fully reflect the complex dynamic conditions of porous media, they provide useful comparative trends.

**Figure 1:**
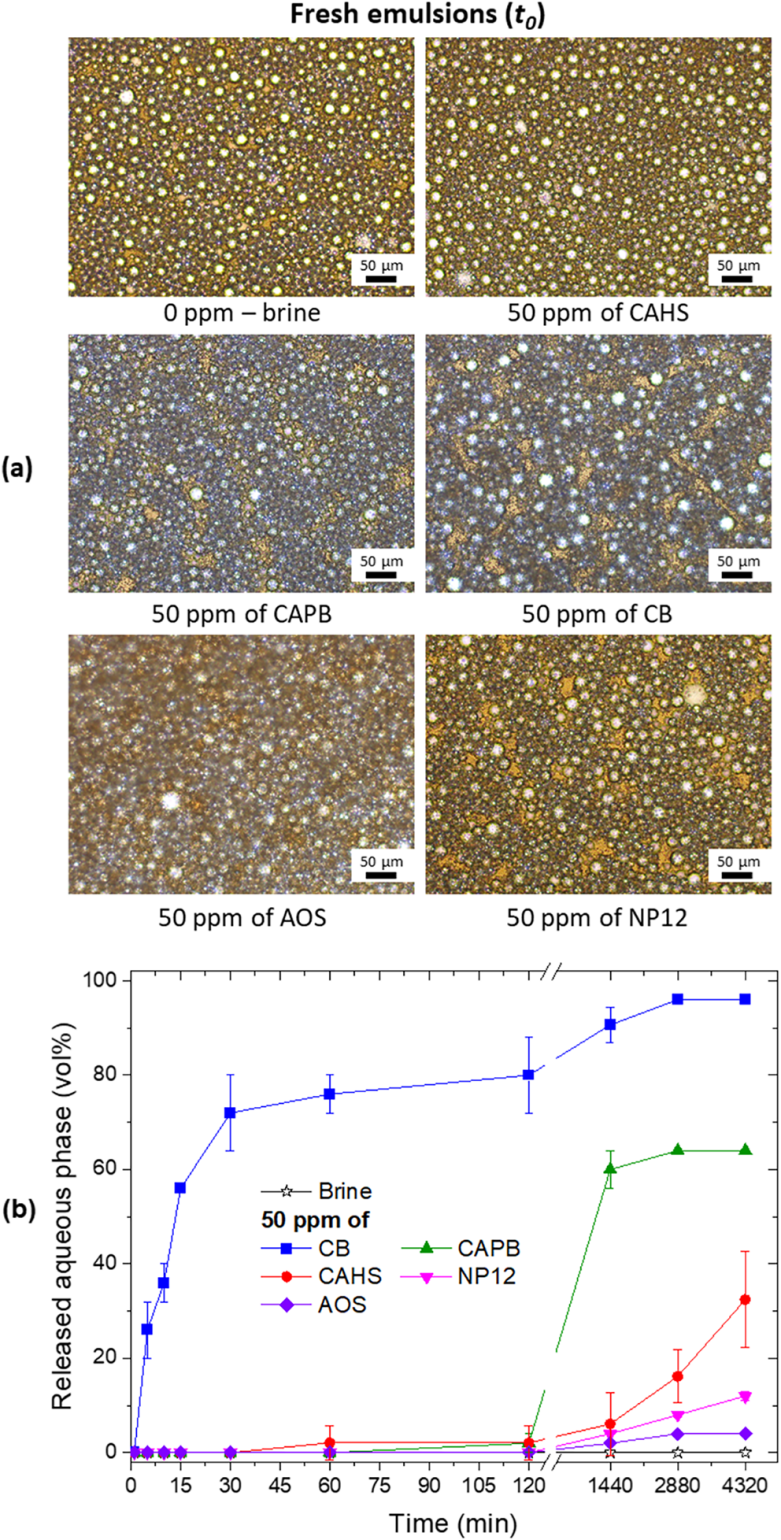
(a) Micrographs of fresh emulsions (0 min—*t*_0_) prepared with 50 ppm of surfactants; and (b) volume fraction of aqueous phase released over time.

CAHS was selected to investigate the mechanism of surfactants in the emulsion phase separation, due to its intermediate influence among all the surfactants tested. Microscopy and water content analyses (Figure 2a) revealed that, while the brine emulsion remained homogeneous, adding 50 ppm of CAHS led to a heterogeneous droplet size distribution after 24 h. During this period, an increase in water content was observed from the top to the bottom of the tube. Although no visible separation occurred after 2 h, the emulsion viscosity with CAHS dropped by around 50% (Figure 2b), indicating the separation process had begun early on. Hence, the surfactant effect followed a typical coalescence and sedimentation process. The posterior addition of CAHS to an emulsion freshly prepared without surfactants caused a similar behavior (Figure S3), confirming that this process was not related to the formation of emulsion but to its destabilization kinetics.

**Figure 2:**
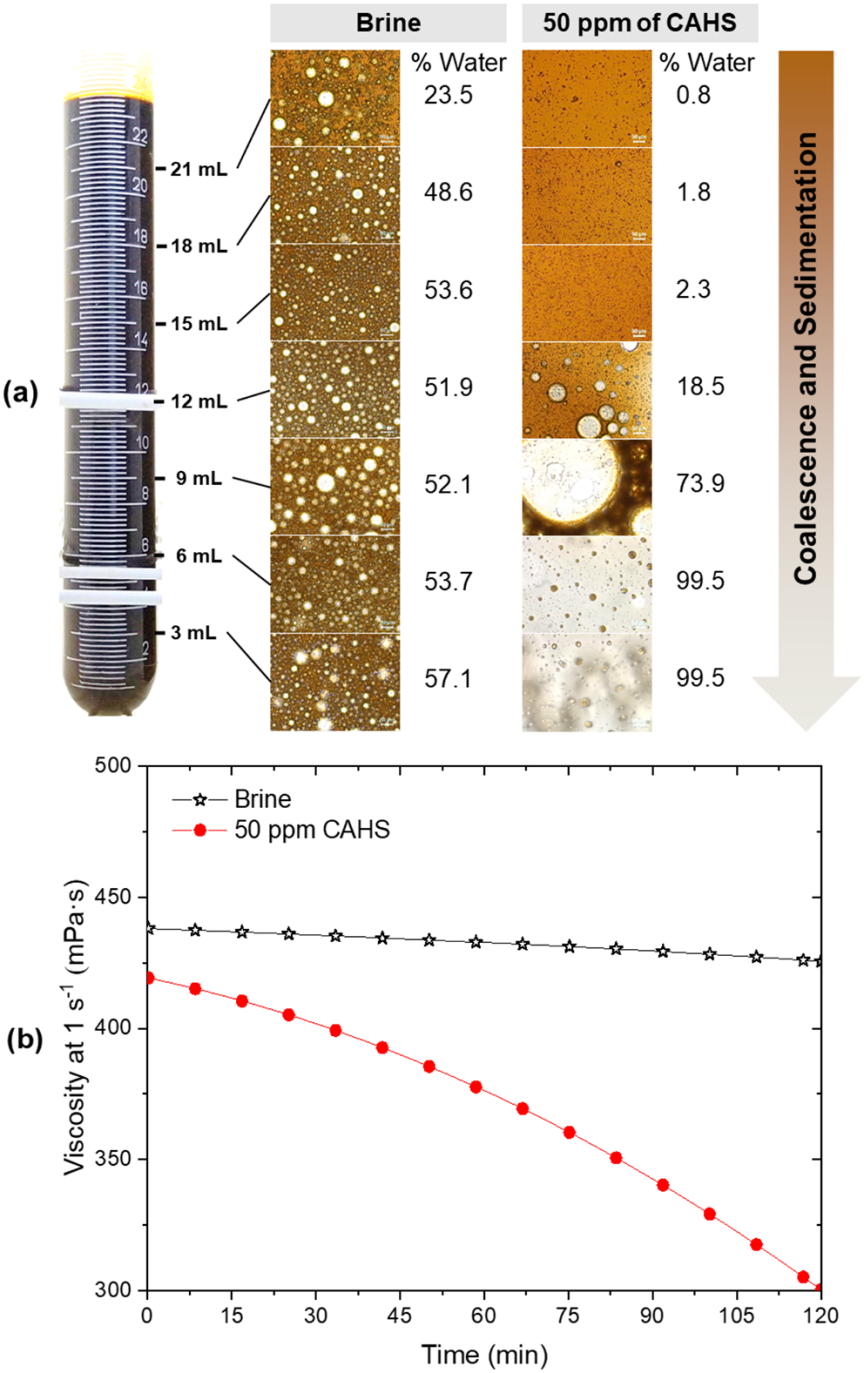
(a) Characteristics of W/O emulsions prepared with 0 and 50 ppm of CAHS after 24 h; and (b) viscosity evolution over 2 h. Water content error <3%.

The destabilization induced by the surfactants can be correlated with their colloidal properties, particularly with the CMC and interfacial activity. The CMC values in brine were below 50 ppm, the concentration used in emulsion preparation (Table 2) (surface tension curves over surfactant concentration in Figure S4). A comparison among the zwitterionic surfactants shows that the lower their CMC in brine, the greater the emulsion destabilization. This trend suggests that hydrophobicity plays an important role because CMC usually decreases with surfactants’ hydrophobicity. However, this trend was not followed by the nonionic and anionic surfactants (NP12 and AOS), which exhibited CMC values similar to that of CAPB, but their impact on emulsion stability was much smaller. Hence, differences in the surfactant’s polar headgroup structure influence their interactions at the oil–brine interface to destabilize it.

**Table 2:** Colloidal Properties of Surfactants and Volume Fraction of Aqueous Phase Released after 72 h for Emulsions with 50 ppm of Surfactants

surfactant	CMC in brine^a^ (ppm)	ΔIFT^b^ (mN·m^–1^)	aqueous phase released after 72 h (vol %)
brine			0
CB	9 ± 1	6.8 ± 0.1	96
CAPB	23 ± 1	3.4 ± 0.1	64
CAHS	49 ± 1	3.3 ± 0.1	40
NP12	25 ± 1	2.2 ± 0.1	12
AOS	20 ± 1	1.4 ± 0.2	4

a Determined by surface tension measurements (Figure S4).

b ΔIFT refers to the difference in IFT between systems with and without surfactant after 5 min at 40 °C.

Dynamic IFT was measured to further investigate the activity of the surfactant at the brine-oil interface. Without surfactant addition, the IFT between crude oil and brine decreased from approximately 14 to 12 mN·m^–1^ over time due to indigenous interfacially active oil compounds. The surfactants significantly reduced the IFT, even at 5 ppm (below CMC) (Figure 3). For CB, measurements could only be performed up to 5 min, as the drop detached from the needle at longer times due to the very low IFT. CAPB and CAHS were measurable up to 10 min, while NP12 and AOS remained stable for over 30 min, consistent with their slower adsorption kinetics. To enable quantitative comparison across all systems, the IFT values at 5 min were used as a reference. The difference in the IFT of systems with and without the surfactants (ΔIFT, Table 2) showed the same trend observed for emulsion destabilization (Figure 1): CB > CAPB ≈ CAHS > NP12 > AOS. Notably, CB reduced IFT to around 5 mN·m^–1^ within 5 min, emphasizing its rapid adsorption at the interface. The faster adsorption of zwitterionic surfactants compared to AOS and NP12 is a key factor in emulsion destabilization and may also account for their potential as W/O demulsifiers. While NP12 and AOS could eventually reach lower IFT values over longer time scales, their slower interfacial adsorption is less effective under the dynamic conditions of emulsification, where viscosity and droplet interactions hinder equilibration.

**Figure 3:**
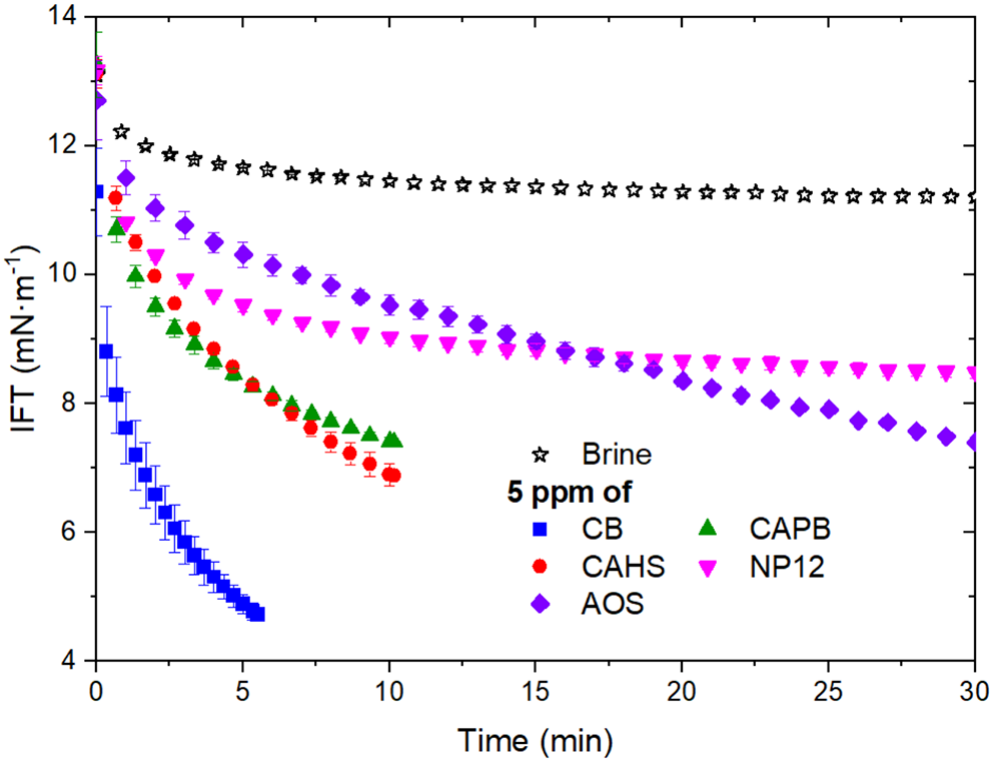
Dynamic IFT between oil and brine in the absence and presence of 5 ppm of surfactants at 40 °C.

It is worth noting that all experiments were conducted with a synthetic seawater brine, which is the standard aqueous medium in offshore oil production. Previous studies have shown that salinity and divalent ions can significantly affect emulsion stability by modifying interfacial film rigidity and droplet interfacial curvature, causing coalescence.^31,32^ Using seawater as the reference system ensured that our results reflected realistic FAWAG conditions, where brine is inherently involved in emulsification.

### 3.2 Impact of Zwitterionic Surfactant Concentration on the Emulsion Formation and Stability

A wider concentration range (100–1000 ppm) was evaluated to better understand the potential impact of zwitterionic surfactants in emulsion formation and stability. These conditions simulate a scenario in which the surfactant concentration in back-produced water rises due to low reservoir adsorption or overdosing. The time necessary to separate 50 vol % of the aqueous phase (*t*_50_) decreased with increasing surfactant concentration (Figure 4a), which can be linked to a faster rate of surfactant adsorption (as evidenced by the rapid IFT reduction of CAHS, Figure S5). However, beyond 500 ppm, the concentration increase led to an unexpected rise in the *t*_50_, i.e., decelerating the phase separation time (full separation kinetics data available in Figure S6). A similar nonmonotonic behavior emerged by plotting data from other studies with cationic and nonionic surfactants, different emulsification protocols, temperature, salinity, and oil components and/or properties^22,24,26,27,29^ (Figure 4b), indicating a general trend that is independent of specific variables. Several hypotheses have been proposed in the literature to explain this type of behavior. For example, the formation of an elastic and viscous film caused by the excess of surfactant,^24^ the inversion of emulsion type from W/O to O/W,^26,27^ and reaching the optimum surfactant dosage.^22,29^ Nevertheless, these studies have not provided robust experimental evidence to support their hypotheses clearly.

**Figure 4:**
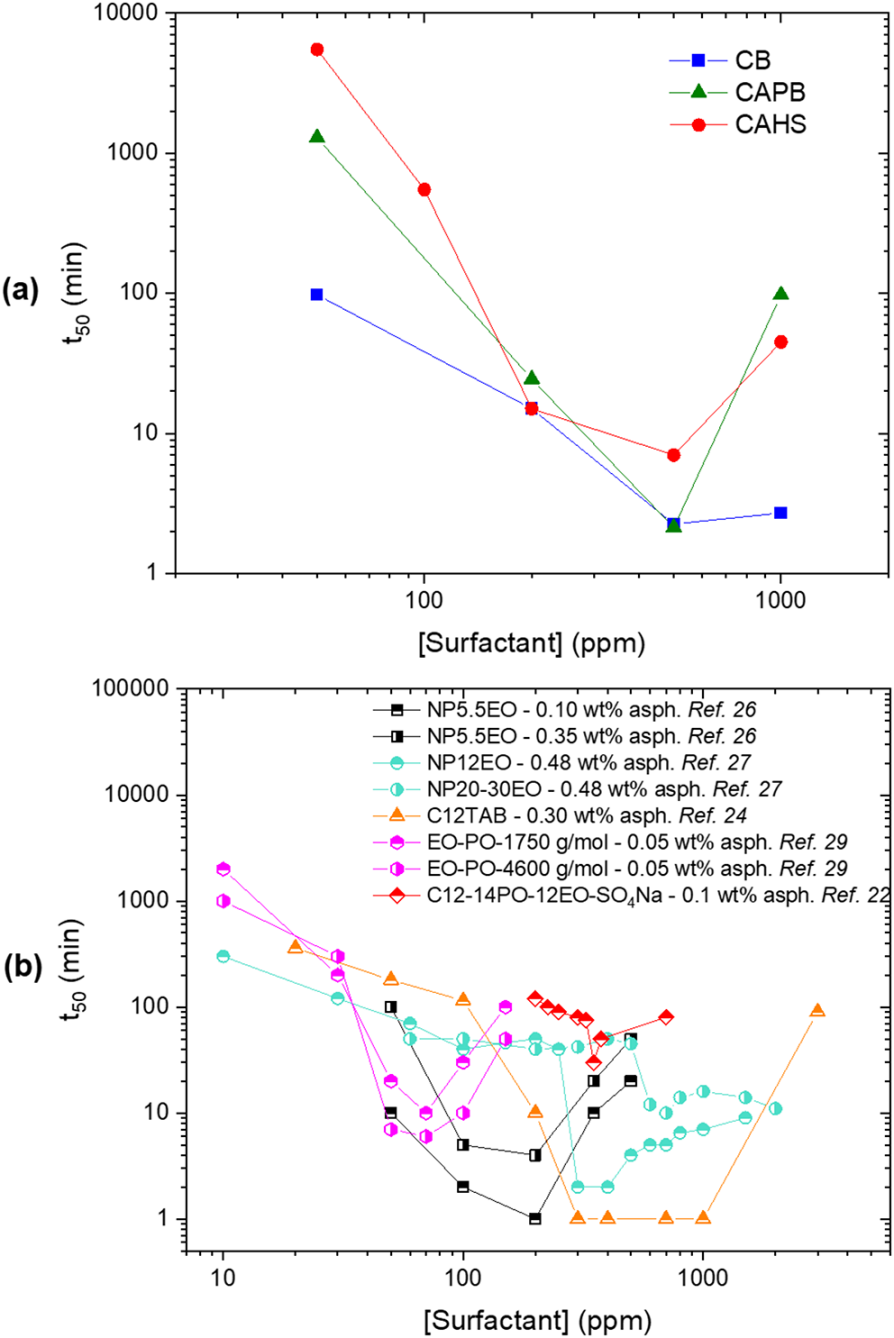
(a) Time to release 50% of the aqueous phase (*t*_50_) as a function of zwitterionic surfactant concentration; and (b) *t*_50_ as a function of surfactant concentration under different conditions reported in the literature, showing nonmonotonic behavior.

Optical microscopy was used in the present study to elucidate the nonmonotonic behavior in more detail (Figure 5) by evaluating droplet size and polydispersity index (PDI) (Table 3) of fresh emulsions prepared at different CAHS concentrations (curves of droplet size distribution are shown in Figure S7). From 0 to 100 ppm of CAHS, the droplets have similar dimensions and PDI, indicating the surfactant did not impact the W/O emulsion formation. At 200 ppm of CAHS, water droplets became 40% larger and three times more polydisperse. At 500 ppm, multiple W/O/W emulsions with large oil droplets were formed, and further increasing the concentration led to a reduction in the size and PDI of the oil droplets. The reduction in droplet size and PDI above 500 ppm of surfactant can be attributed to the increased surfactant availability, which improves the coverage of the oil–water interface, resulting in smaller droplets. A similar trend, including the formation of W/O/W emulsions, was observed for CB and CAPB zwitterionic surfactants (Figure S8). Conductivity measurements confirm this emulsion inversion, showing very low conductivity for oil-continuous systems and values up to 8 orders of magnitude higher for water-continuous systems (Table 3).

**Figure 5:**
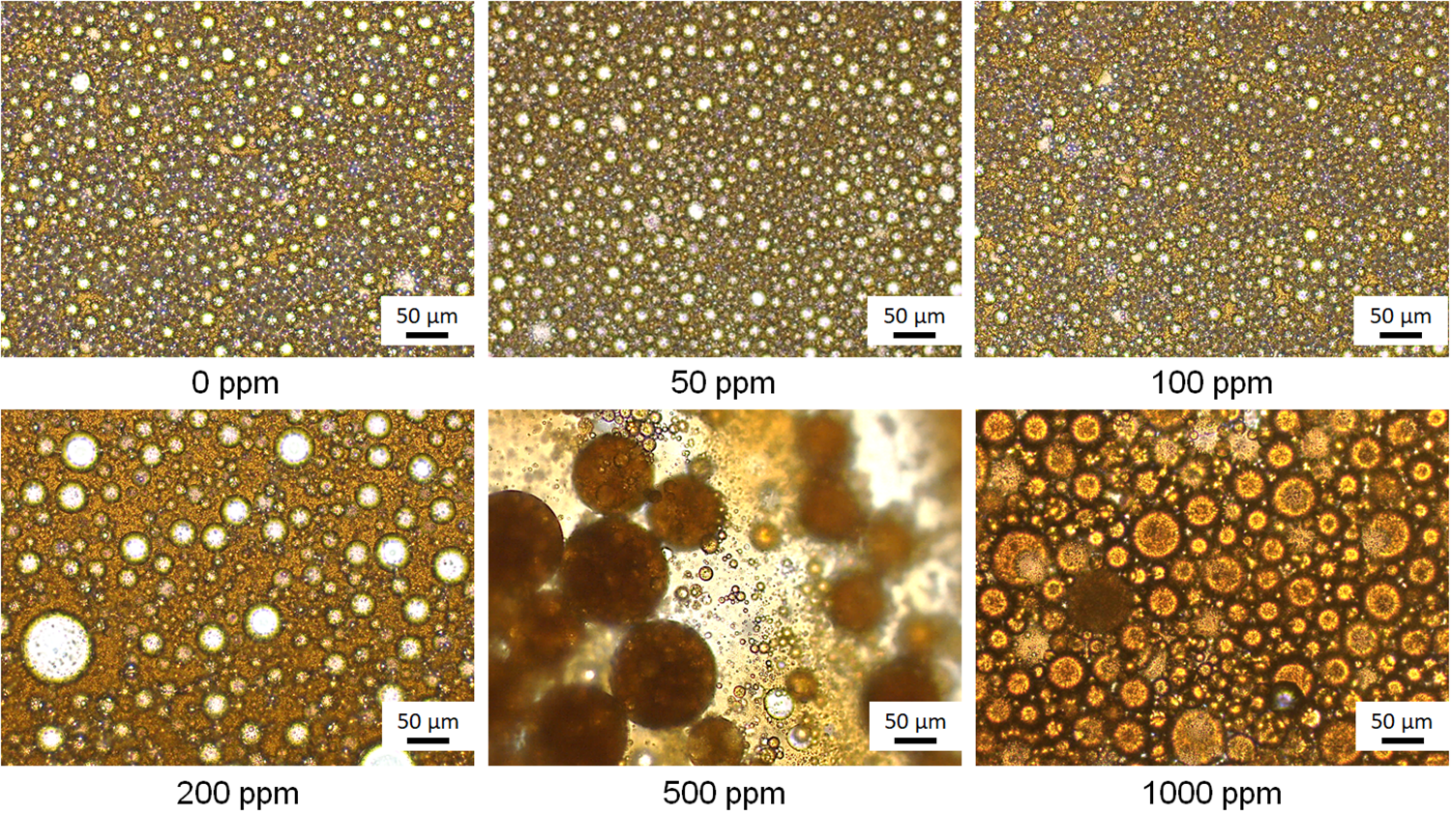
Micrographs of fresh emulsions prepared with increasing concentrations of CAHS.

**Table 3:** Emulsion Type, Average Droplet Size, and Polydispersity Index (PDI) for Fresh Emulsions Prepared with Different CAHS Concentrations

[CAHS] (ppm)	emulsion type	*D*_1,0_ (μm)	PDI	conductivity (μS cm^–1^)
0	W/O	10 ± 5	0.26	
50	W/O	11 ± 6	0.27	6.0 × 10^–6^
100	W/O	11 ± 5	0.22	
200	W/O	15 ± 13	0.75	1.2 × 10^–5^
500	W/O/W	57 ± 51^a^	0.82	
1000	W/O/W	27 ± 16^a^	0.36	5.9 × 10^3^

a diameter of oil droplets.

Based on these results, the dependence of stabilization and inversion mechanisms on zwitterionic surfactant concentration was proposed and illustrated in Figure 6. Hydrophilic surfactants induce a curvature inversion of the emulsion droplets as the concentration increases, as reported by Fan et al.^28^ However, zwitterionic surfactants induced a faster destabilization than NP12 and AOS. This difference can be attributed to specific interactions between the betaine headgroups and crude oil components at the interfacial film. Figure 6 shows that the W/O emulsions prepared without surfactants remained stable even after 72 h due to indigenous interfacially active oil compounds. At low concentrations (up to 100 ppm of CAHS, for example), the zwitterionic surfactants did not affect the emulsion formation but destabilized the formed W/O emulsions. As the concentration continued to increase (200 ppm of CAHS), the amount of surfactant molecules was sufficient to increase the initial water droplet sizes, reducing their curvature. At high concentrations (above 500 ppm of CAHS), multiple W/O/W emulsions formed, with internal interfaces dominated by indigenous oil surfactants and external interfaces by CAHS molecules. Moreover, further increasing the surfactant concentration (>1000 ppm) reduced O/W droplet size. This reduction of oil droplets in the aqueous phase also indicates enhanced stability of O/W emulsions, which could be a concern for the produced water quality if a high concentration of foaming surfactant is produced.

**Figure 6:**
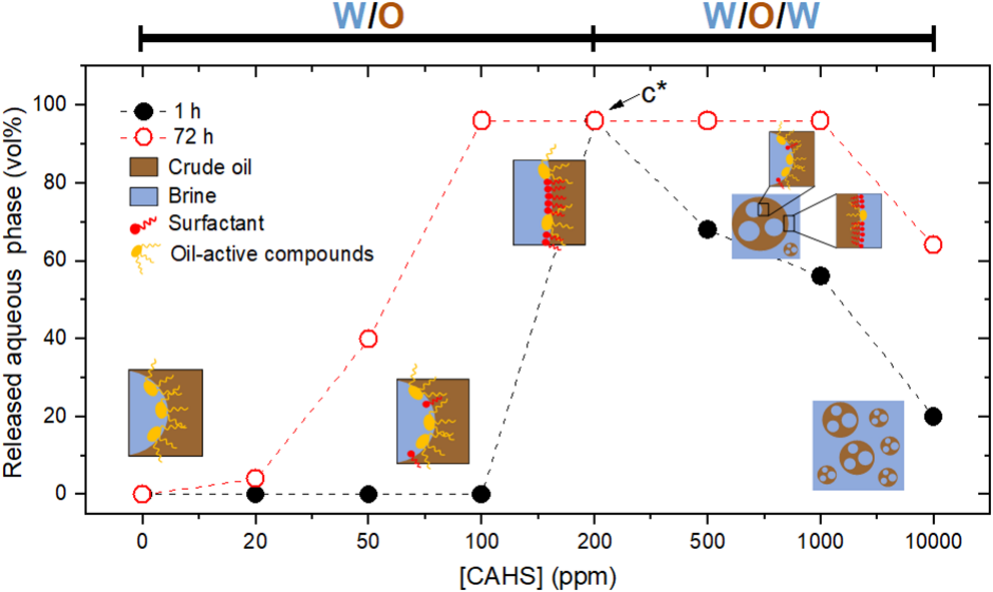
Dependence of stabilization and inversion mechanisms on surfactant concentration. c* is the critical concentration: below it, W/O emulsions form; above it, W/O/W emulsions appear.

### 3.3 Impact of Zwitterionic Surfactants on Water Quality

The oil content in the aqueous phases, separated after 72 h, was used to evaluate the impact of zwitterionic surfactant on water quality. The oil content increased from 46 ppm in brine to at least 102 ppm in the presence of 50 ppm of surfactants (Figure 7a). Among the surfactants tested, CB exhibited the most pronounced effect, likely due to its longer carbon tail, which enhances hydrophobicity and interfacial activity. Therefore, even at low residual concentrations, surfactants adversely affected the produced water quality.

**Figure 7:**
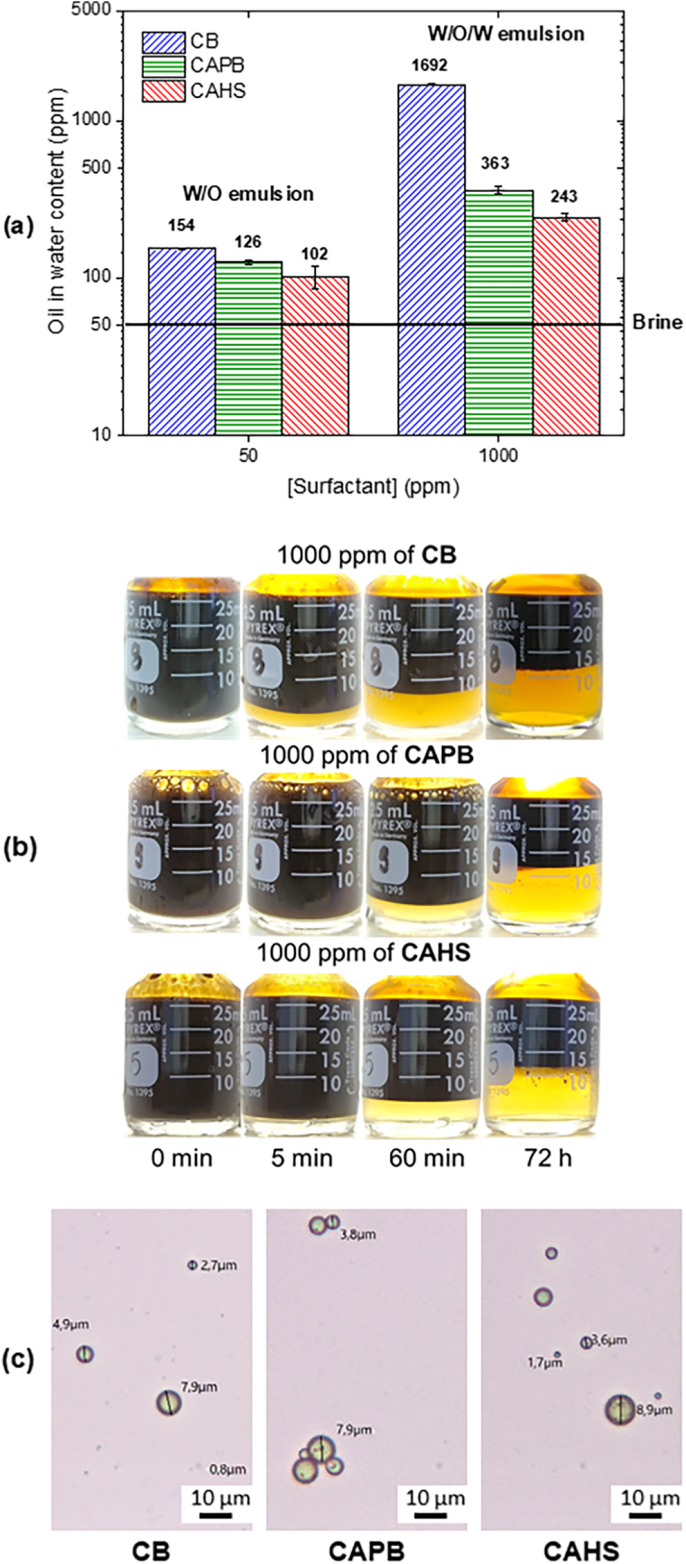
(a) Oil-in-water content after 72 h for emulsions prepared using brine (black line), 50 and 1000 ppm of surfactants; (b) bottle test results over time for the emulsion with 1000 ppm of surfactants; and (c) micrographs of the aqueous phase of emulsions with 1000 ppm of surfactants after 72 h, showing oil-in-water (O/W) droplets.

Increasing the concentration of CAHS to 200 ppm, the oil content in the separated aqueous phase remained relatively unchanged (103 and 115 ppm, respectively, Figure S9). A more pronounced influence in water quality was observed at 1000 ppm, where emulsion inversion occurred (Figure 7a). Over 72 h, the W/O/W multiple emulsions separated into two macroscopic phases (aqueous-rich and oil-rich), except at very high concentrations, such as 10,000 ppm (Figure S10). The respective aqueous-rich phases became more colorful over time because of this W/O/W emulsion separation (Figure 7b). After 72 h, this phase consisted mainly of oil-in-water dispersions (Figure 7c). At 1000 ppm, CB showed a notably higher oil content in water and a marked color difference compared to CAPB and CAHS, reinforcing the influence of hydrophobicity and interfacial properties on surfactant behavior.

To the best of our knowledge, this decline in produced water quality induced by zwitterionic surfactants has not been previously reported. This finding is particularly relevant since higher oil content in produced water can lead to increased operational costs due to additional water treatment steps. Moreover, it could challenge compliance with environmental regulations concerning the maximum allowed oil content for water disposal. Thus, these findings underscore the importance of monitoring the back-production of zwitterionic surfactant in EOR processes.

## 4 Conclusion

The interfacial activity of zwitterionic surfactants used for foam stabilization in porous media can significantly impact water–oil separation during upstream oil processing. On the one hand, they promote phase separation more effectively than typical anionic and nonionic surfactants at residual concentrations. On the other hand, high concentrations can worsen the quality of produced water and induce W/O/W emulsions. To the best of our knowledge, this is the first report of W/O/W emulsification involving zwitterionic surfactants and crude oil. These multiple emulsions can also compromise the kinetics of water–oil separation. An optimal concentration of 200 ppm was identified under the studied conditions, providing rapid aqueous phase separation kinetics while maintaining relatively low oil content (around 100 ppm) in the separated water. Above this optimal concentration, the quality of the produced water significantly declined. A comparison among the zwitterionic surfactants used in this work highlights the critical role of surfactant hydrophobicity. The surfactant with the longest carbon chain CB, promoted faster phase separation of W/O emulsions, but led to significantly increased oil contamination in the produced water. These findings highlight the need for careful selection of foaming surfactants, and for rigorous monitoring of their concentrations in FAWAG-EOR applications, to ensure that aqueous phase separation can be achieved without adverse operational, economic, or environmental impacts.

## Supplementary Material



## Notes

Crude oil properties (Table S1); calibration curve for oil content in aqueous phase (Figure S1); droplet size distribution of the fresh emulsions prepared with different surfactants (Figure S2); aqueous phase release over time for emulsions with 50 ppm of CAHS, prepared before and after homogenization (Figure S3); CMC curves for CAHS, CAPB, CB, AOS, and NP12 (Figure S4); dynamic IFT curves for crude oil–brine systems with 2–20 ppm of CAHS (Figure S5); aqueous phase release over time for emulsions formed with of CB, CAPB, and CAHS varying surfactant concentrations (Figure S6); droplet size distribution of the fresh emulsions prepared with different concentrations of CAHS (Figure S7); emulsion type for different concentrations of CAPB and CB (Figure S8); oil-in-water content after 72 h for emulsions with 0–1000 ppm of CAHS (Figure S9); emulsions prepared with 10000 ppm of CAHS after 72 h (Figure S10) (PDF)

## Nomeclature/Abbreviation

AOS: sodium olefin sulfonate C14–C16
CAHS: cocamidopropyl hydroxysultaine
CAPB: cocamidopropyl betaine
CB: cetyl betaine
CMC: critical Micelle Concentration
EOR: enhanced Oil Recovery
FAWAG: foam assisted water alternating gas
HLB: hydrophilic lipophilic balance
IFT: interfacial tension
NP12: nonylphenol ethoxylate with 12 EO
O/W: oil-in-water emulsion
PDI: polydispersity index
*t*_50_: time to release 50% of aqueous phase
W/O: water-in-oil emulsion
W/O/W: water-in-oil-in-water multiple emulsion
ΔIFT: difference in interfacial tension between systems with and without surfactant after 5 min.
