# Supplementary material for: Impact of Residual Zwitterionic Surfactants on Topside Water–Oil Separation of Pre-Salt Light Crude Oil Emulsions
Source: ACS Omega. 2025 Oct 14;10(42):50340–8. doi: 10.1021/acsomega.5c07388 (PMC12573017; doi:10.1021/acsomega.5c07388)
Supplement: Supplementary file 1 [file ao5c07388_si_001.pdf]

# Supplementary Information

## Impact of Residual Zwitterionic Surfactants on Topside Water-Oil Separation of Pre-Salt Light Crude Oil Emulsions

Bruno G. Alvarenga[a], Angela C. P. Duncke[a], Aurora Pérez-Gramatges[a,b], and Ana M Percebom[b]

[a] *Laboratory of Surfactant Physical-Chemistry (LASURF), Pontifical Catholic University of Rio de Janeiro, PUC-Rio, Rio de Janeiro, RJ 22451-900, Brazil*

[b] *Department of Chemistry, Pontifical Catholic University of Rio de Janeiro, PUC-Rio, Rio de Janeiro, RJ 22451-900, Brazil*

**Corresponding author: apercebom@puc-rio.br*

**Table S1.** Properties of the Brazilian pre-salt crude oil.

| | | |
|---|---|---|
| **ρ** ($g/cm^3$) | 40 °C | 0.872 |
| | 65 °C | 0.855 |
| **η** (mPa.s) | 40 °C | 21 |
| | 65 °C | 10 |
| **Saturates** (wt%) | | 41.0 ± 1.6 |
| **Aromatics** (wt%) | | 32.0 ± 1.1 |
| **Resins** (wt%) | | 15.3 ± 0.3 |
| **Asphaltenes** (wt%) | | < 0.5 (≈ 0.1) |

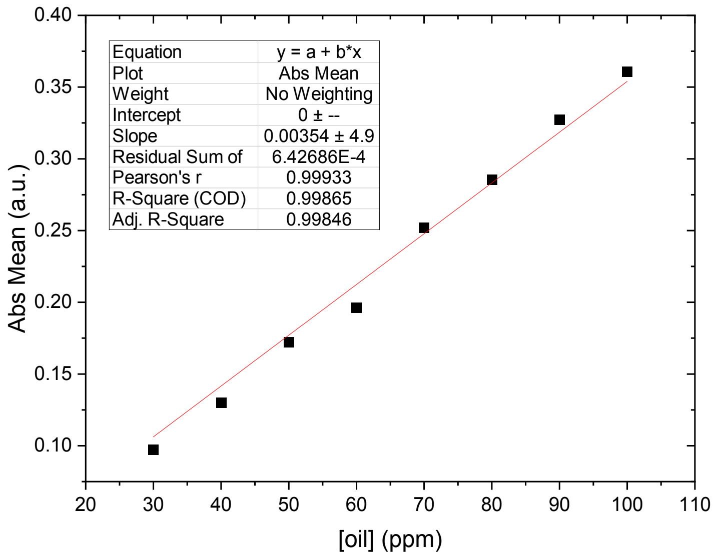


**Figure S1.** Calibration curve obtained with 30–100 ppm of oil in chloroform at 400 nm, used to determine oil-in-water content.

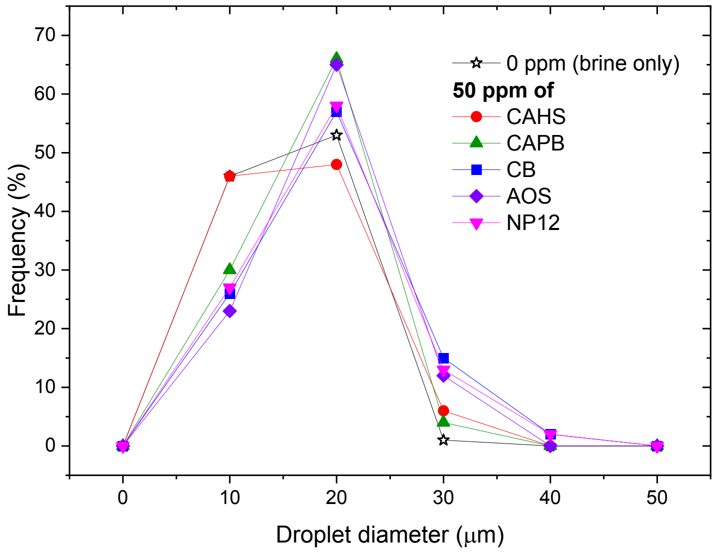


**Figure S2.** Droplet size distribution of the fresh emulsions prepared with no surfactant (brine only) and the surfactants tested CAHS, CAPB, CB, AOS, and NP12, at 50 ppm.

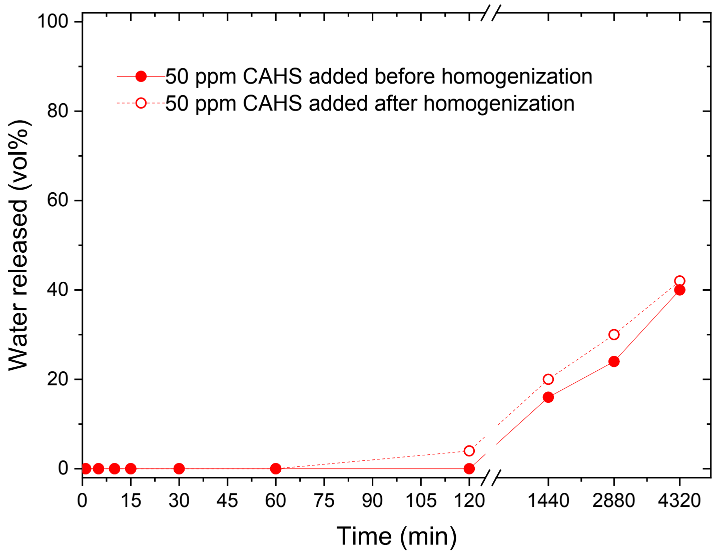


**Figure S3.** Volume fraction of aqueous phase released over time for emulsions with 50 ppm of CAHS, prepared before and after homogenization. Adding CAHS to an emulsion freshly prepared without surfactants caused the same phase separation as when CAHS was added to the aqueous phase before emulsification, indicating its effect on destabilization kinetics but not emulsion formation.

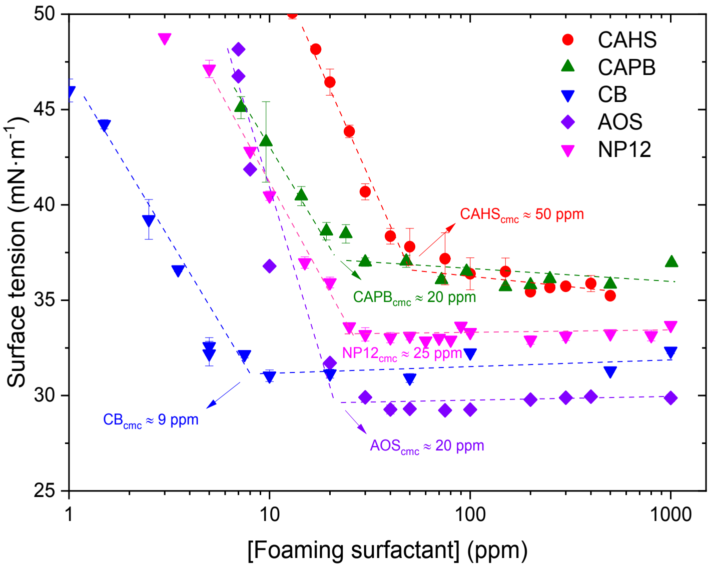


**Figure S4.** Surface tension curves to determine critical micelle concentration (CMC) for CAHS, CAPB, CB, AOS, and NP12, as indicated by arrows.

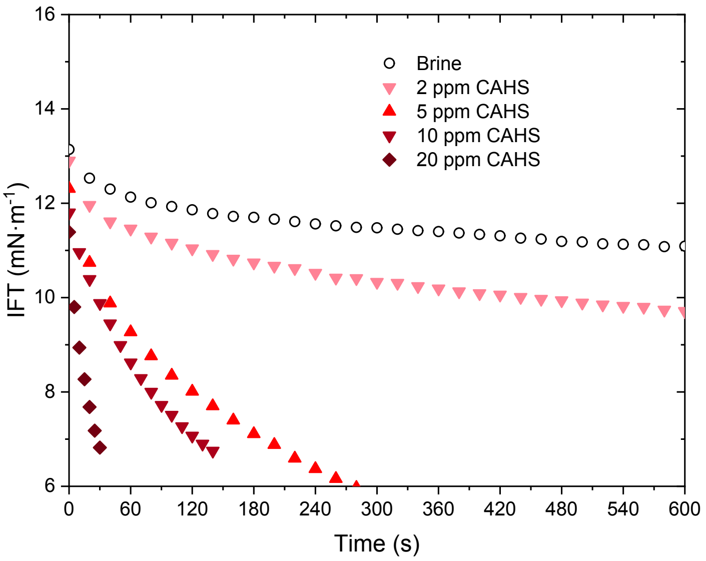


**Figure S5.** Dynamic interfacial tension (IFT) curves between crude oil and brine, without and with 2–20 ppm of CAHS.

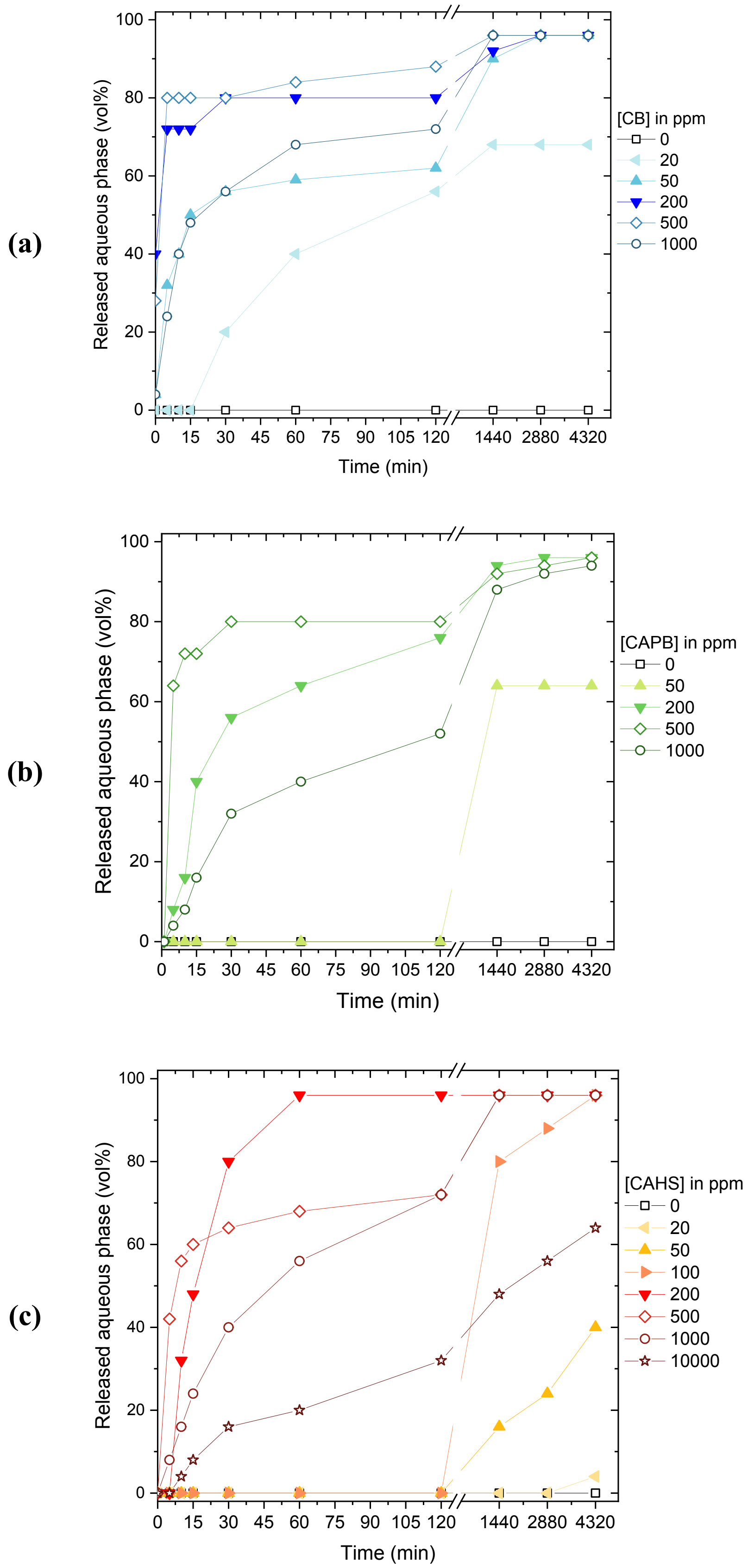


**Figure S6.** Volume fraction of aqueous phase released over time for emulsions with different surfactant concentrations: (a) CB, (b) CAPB, and (c) CAHS.

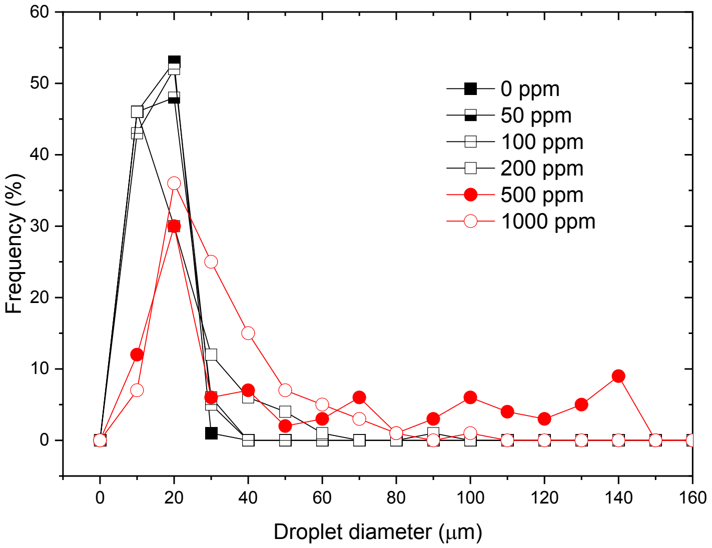


**Figure S7.** Droplet size distribution of the fresh emulsions prepared with different concentrations of CAHS surfactant. Black curves are related of W/O systems (up to 200 ppm) while red curves are related to oil droplet size from the W/O/W multiple emulsions.

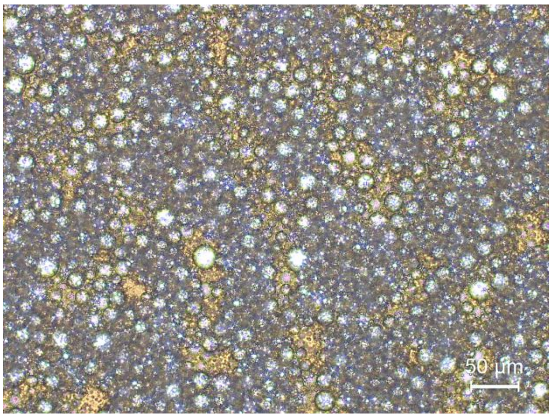


**50 ppm of CAPB**

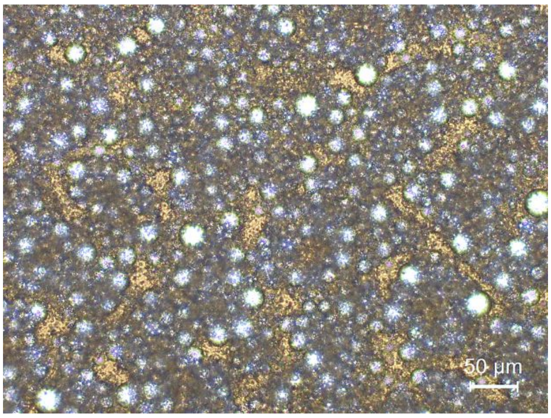


**50 ppm of CB**

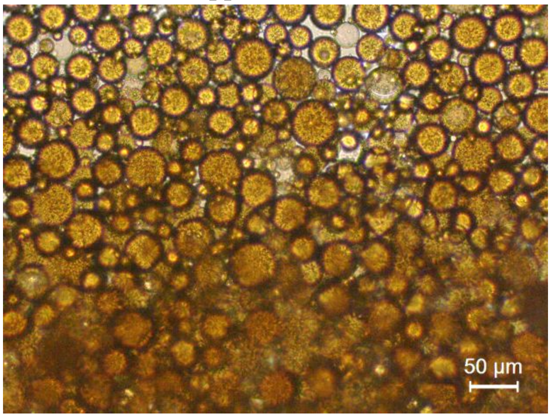


**1000 ppm of CAPB**

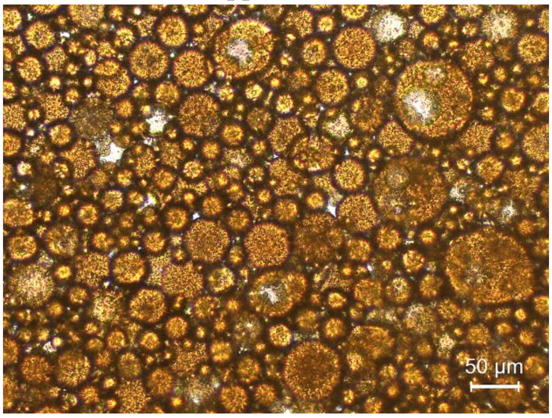


**1000 ppm of CB**

**Figure S8.** Micrographs of fresh emulsions prepared with different concentrations of CAPB and CB, showing a transition from water-in-oil (W/O) to water-in-oil-in-water (W/O/W) emulsions.

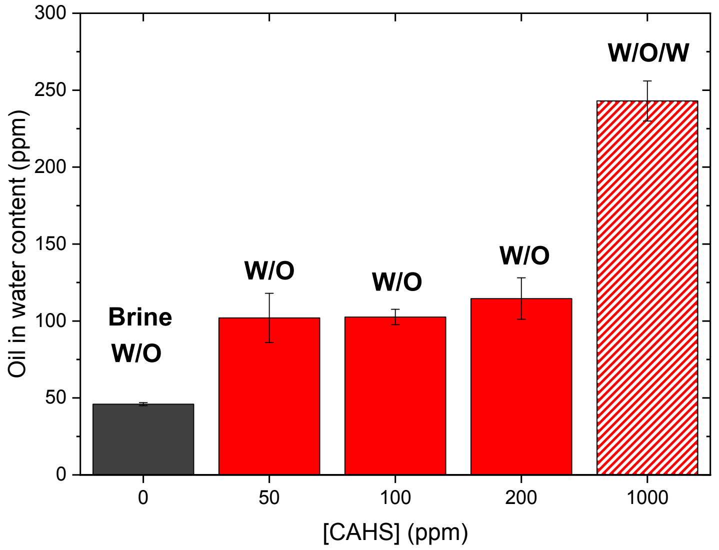


**Figure S9.** Oil-in-water content after 72 hours for emulsions prepared with (a) 0, 50, 100, 200, and 1000 ppm of CAHS.

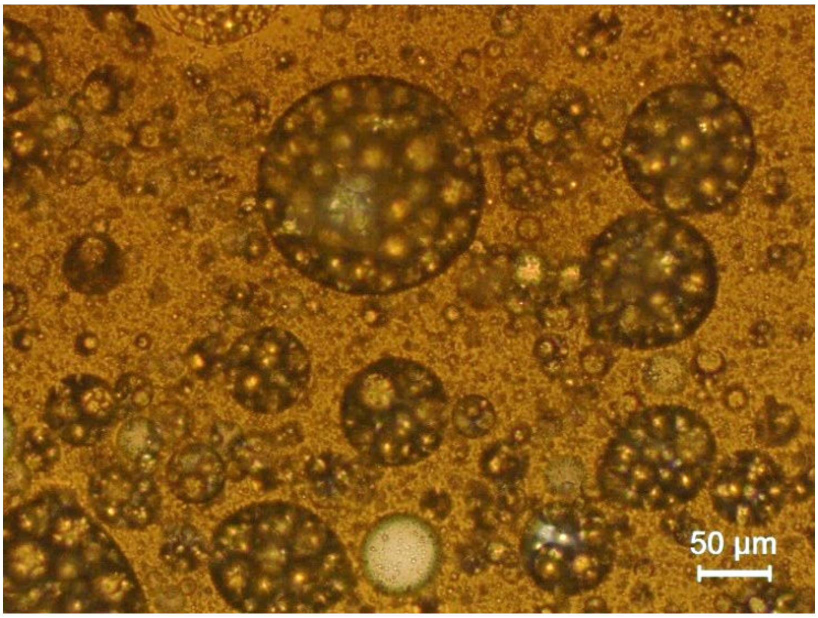


**Figure S10.** Micrographs of emulsions with 10,000 ppm of CAHS after 72 h, showing the persistence of W/O/W multiple emulsions.
